# TAM-associated CASQ1 mutants diminish intracellular Ca^2+^ content and interfere with regulation of SOCE

**DOI:** 10.1007/s10974-024-09681-9

**Published:** 2024-08-10

**Authors:** Alessandra Gamberucci, Claudio Nanni, Enrico Pierantozzi, Matteo Serano, Feliciano Protasi, Daniela Rossi, Vincenzo Sorrentino

**Affiliations:** 1 Department of Molecular and Developmental Medicine, University of Siena, Siena, I-53100 Italy; 2 Center for Advanced Studies and Technology, CAST, University G. d’Annunzio of Chieti-Pescara, Chieti, I-66100 Italy; 3 DMSI, Department of Medicine and Aging Sciences, University G. d’Annunzio of Chieti-Pescara, Chieti, I-66100 Italy; 4 Interdepartmental Program of Molecular Diagnosis and Pathogenetic Mechanisms of Rare Genetic Diseases, Azienda Ospedaliero Universitaria Senese, Siena, I-53100 Italy

**Keywords:** Store operated calcium entry, Skeletal muscle, Myopathy, Calcium, Sarcoplasmic reticulum

## Abstract

Tubular aggregate myopathy (TAM) is a rare myopathy characterized by muscle weakness and myalgia. Muscle fibers from TAM patients show characteristic accumulation of membrane tubules that contain proteins from the sarcoplasmic reticulum (SR). Gain-of-function mutations in STIM1 and ORAI1, the key proteins participating in the Store-Operated Ca^2+^ Entry (SOCE) mechanism, were identified in patients with TAM. Recently, the *CASQ1* gene was also found to be mutated in patients with TAM. CASQ1 is the main Ca^2+^ buffer of the SR and a negative regulator of SOCE. Previous characterization of CASQ1 mutants in non-muscle cells revealed that they display altered Ca^2+^dependent polymerization, reduced Ca^2+^storage capacity and alteration in SOCE inhibition. We thus aimed to assess how mutations in CASQ1 affect calcium regulation in skeletal muscles, where CASQ1 is naturally expressed. We thus expressed CASQ1 mutants in muscle fibers from *Casq1* knockout mice, which provide a valuable model for studying the Ca^2+^ storage capacity of TAM-associated mutants. Moreover, since *Casq1* knockout mice display a constitutively active SOCE, the effect of CASQ1 mutants on SOCE inhibition can be also properly examined in fibers from these mice. Analysis of intracellular Ca^2+^ confirmed that CASQ1 mutants have impaired ability to store Ca^2+^and lose their ability to inhibit skeletal muscle SOCE; this is in agreement with the evidence that alterations in Ca^2+^entry due to mutations in either STIM1, ORAI1 or CASQ1 represents a hallmark of TAM.

## Introduction

Calsequestrin (CASQ) is an acidic protein of the sarcoplasmic reticulum (SR), with a high capacity, medium-low affinity for Ca^2+^ (MacLennan et al. 1971; Damiani et al. 1990). In the mammalian genome, two CASQ genes are present: CASQ1 and CASQ2, which are expressed in fast- and slow-twitch muscle fibers, and in slow-twitch muscle fibers and cardiac muscle, respectively (Biral et al. 1992). Thanks to its high Ca^2+^ binding capacity, CASQ represents the main buffer system of the SR that allows the storage and accumulation of the large amount of Ca^2+^ necessary for muscle contraction, reaching up to 80 nmol Ca^2+^/nmol protein (Park et al. 2004). Structural and functional studies have revealed that Ca^2+^ binding is a very dynamic process, based on Ca^2+−^dependent conformational changes in CASQ that assembles in large ribbon-like polymers, that preferentially accumulate within the terminal cisternae of the SR (Franzini-Armstrong et al. 1987). Here, two transmembrane proteins, triadin and junctin anchor polymers of CASQ in proximity of the ryanodine receptors (RyRs), the Ca^2+^ release channels of the SR, forming a regulated quaternary complex that support Ca^2+^ release during the excitation contraction coupling (ECC) mechanism (Zhang et al. 1997; Kobayashi et al. 2000; Shin et al. 2000; Glover et al. 2002; Rossi et al. 2014a; Beard and Dulhunty 2015). Accordingly, studies on mice knockout for *Casq1* revealed that they show a general reduction in SR Ca^2+^ release following either low frequency electrical stimulation or caffeine treatment and a significant impairment in sustaining prolonged muscle activity suggesting a severe reduction in Ca^2+^ storage capacity (Paolini et al. 2007; Tomasi et al. 2012). More interestingly, structural studies revealed that these mice show a profound remodeling of the SR and the T-tubules, with the appearance of multilayered junctions. Although this was originally explained by the development of a compensatory effect in response to CASQ1 reduction, more recently it has been proposed that these structures might represent Calcium Entry Units (CEU), where extracellular calcium enter the muscle fiber to refill SR stores via the Store Operated Ca^2+^ Entry (SOCE) mechanism (Michelucci et al. 2022). Muscle contraction requires repetitive activation of ECC to sustain prolonged muscle activity; this long-lasting stimulation may result in an elevation of cytoplasmic Ca^2+^ levels for prolonged periods, a condition that may cause extrusion of some Ca^2+^ ions by the activity of Na^+^/Ca^2+^ exchangers and/or plasma membrane Ca^2+^-ATP-ases (PMCA) (Gissel and Clausen 2000; Overgaard et al. 2004; Allen et al. 2008; Pearce et al. 2023). In *Casq1* knockout mice, constitutive activation of SOCE is required to counteract Ca^2+^ loss favoring refilling of the depleted SR stores (Michelucci et al. 2022). Two main players are known to act in SOCE: the stromal-interacting molecule-1 (STIM1), an SR resident protein that functions as a Ca^2+^ sensor and the Ca^2+^ release-activated calcium channel protein 1 (Orai1), a Ca^2+^ release-activated channel (CRAC) in the plasma membrane, which opens upon interaction with STIM1 (Roos et al. 2005; Feske et al. 2006). More recently, in addition to its role as a SR Ca^2+^ buffer, CASQ1 was shown to play a role as a negative regulator of SOCE, by binding both STIM1 and STIM2, indicating that, in skeletal muscle, it represents a dual regulator of both ECC and SOCE (Wang et al. 2015; Zhang et al. 2016; Jeong et al. 2021).

According to their role in muscle physiology, mutations in *STIM1*, *ORAI1* and *CASQ1* are associated with defects in Ca^2+^ homeostasis that result in human congenital myopathies. Recessive loss-of-function mutations in *STIM1* and *ORAI1* are associated with muscular hypotonia and severe immunodeficiency, this second aspect due to the key role of SOCE in the immune system (McCarl et al. 2009; Lacruz and Feske 2015; Choi et al. 2019). Dominant gain-of-function mutations in *STIM1* and *ORAI1* are linked to development of tubular aggregate myopathy (TAM) and Stormorken syndrome (Böhm et al. 2013, 2017; Misceo et al. 2014; Nesin et al. 2014; Böhm and Laporte 2018). Stormorken syndrome manifests as a severe multisystem disorder with symptoms such as miosis, ichthyosis, short stature, hyposplenism, thrombocytopenia, dyslexia, muscle weakness, and intellectual disability. Conversely, TAM is characterized by muscle weakness, myalgia, cramps, elevated creatine kinase levels, and histologically, by the presence of typical packed membrane tubules containing proteins from the SR (Feske et al. 2006; Silva-Rojas et al. 2020). Four mutations in *CASQ1*, (CASQ1^Asp44Asn^, CASQ1^Asn56Tyr^ CASQ1^Gly103Asp^ and CASQ1^Ile385Thr^) have been identified in patients with TAM. Alteration of CASQ1 polymerization is a typical trait of these mutations, resulting in a reduced ability to store Ca^2+^ (Barone et al. 2017; Böhm et al. 2018b). Interestingly, in vitro studies in cultured cell lines, showed that two of these mutants (CASQ1^Asp44Asn^, and CASQ1^Ile385Thr^) lost their ability to inhibit SOCE, mirroring SOCE hyperactivation observed in skeletal muscles of patients expressing gain-of-function mutations in *STIM1* or *ORAI1*; on the contrary, the CASQ1^Gly103Asp^ mutant was still able to inhibit Ca^2+^ influx (Barone et al. 2017).

To further characterize the effect of CASQ1 mutations on Ca^2+^ homeostasis and SOCE in skeletal muscle fibers, we expressed GFP-tagged CASQ1 mutants in Flexor Digitorum Brevis (FDB) muscles from *Casq1* knockout mice. As previously described, *Casq1* knockout mice show a significant decrease in the SR Ca^2+^, indicating that it represents a valuable model to evaluate the effect of CASQ1 mutations on Ca^2+^ store content (Paolini et al. 2007; Tomasi et al. 2012). Moreover, they partially mirror the condition observed in muscle fibers of patients with TAM carrying gain of function mutations in STIM1 and ORAI1, who show a constitutive activation of SOCE; they thus represent a good model to study restoration of CASQ1-dependent SOCE inhibition (Michelucci et al. 2022).

The results obtained show that expression of CASQ1 mutants impairs the loading capacity of the SR and increases SOCE supporting the idea that STIM1, ORAI1 and CASQ1 contribute to regulate the same cellular pathway in skeletal muscle and that alterations in Ca^2+^ entry represent a hallmark of TAM.

## Materials and methods

### Animal care

All procedures implying animals were carried out with the utmost care to minimize animal suffering. Mice were humanely euthanized by CO_2_ overdose, following the guidelines approved by the Animal Care Committee of the University of Siena and in compliance with the regulations set forth by the Italian Ministry of Health (64/2020-PR). These procedures adhere to the standards outlined in Directive 2010/63/EU of the European Parliament and the Council of 22 September 2010 concerning the welfare of animals used for scientific purposes. Furthermore, the study is reported in accordance with the ARRIVE guidelines (https://arriveguidelines.org). Experiments were conducted using adult (2–4 months old) *Casq1* knockout mice (Paolini et al. 2007), which were provided with *ad libitum* access to food and water. The mice were housed under controlled conditions, with a room temperature maintained between 21 and 25 °C and a relative humidity of 50–60%. The light-dark cycle was set to 12 h.

### DNA transfection and FDB muscle fiber isolation

Plasmids expressing GFP-tagged human CASQ1^WT^, CASQ1^Asp44Asn^, CASQ1^Gly103Asp^ and CASQ1^Ile385Thr^ have been previously described (Barone et al. 2017). Transcription of genes coding for GFP-fusion proteins was driven by the human cytomegalovirus (CMV) immediate early promoter and enhancer. CASQ1 proteins from *Homo sapiens* and *Mus musculus* display a 94.69% identity and 96.71% similarity, in addition Asp44, Gly103 and Ile385 amino acids are conserved between species. Transfection of expression vectors was performed as described (Rossi et al. 2019). Briefly, mice were anesthetized with 3% isoflurane and FDB muscle was injected with 5 µl of 2 mg/ml hyaluronidase. 1 h after injection of hyaluronidase, FDB muscle was injected with 10 µg of DNA. Two electrodes were positioned on either side of the muscle, and electric pulses were administered. A series of twenty 120 V electric pulses, each lasting 20 milliseconds with 1-second intervals, were delivered using an electric pulse generator (Electro Square Poraton ECM830; BTX-Genetronics). Mice were sacrificed 10–12 days after electroporation.

Mechanical isolation of muscle fibers from electroporated FDB muscles was performed as described in (Blaauw et al. 2012). FDB muscles were digested with 0.2% Collagenase (Clostridium hystoliticum Type I, Sigma), for 1 h and 30 min at 37 °C/5% CO_2_ and mechanically isolated in Tyrode buffer solution (NaCl 134 mM, KCl 2.68 mM, CaCl_2_ 1.8 mM, MgCl_2_ 1.05 mM, NaH_2_PO_4_ 0.417 mM, NaHCO_3_ 11.9 mM, Glucose 5.56 mM, pH 7.4) added with 10% heat inactivated fetal bovine serum (FBS) (Sigma-Aldrich).

Isolated FDB fibers were plated on Lab Tek™ II four-well plates cover glass (Merk), previously coated with 0.025% laminin (Merk) and analyzed within 24 h. Experiments were performed on selected fibers from at least 3 to 5 mice for each CASQ1-GFP expression vector.

### Measurement of SR Ca^2+^ stores

Isolated FDB fibers were loaded with 5 µM Fura-2 AM (Invitrogen) for 30 min at room temperature in Ringer solution (145 mM NaCl, 5 mM KCl, 1mM MgCl_2_, pH 7,4) containing 2 mM Ca^2+^ and 1% Bovine Serum Albumine (BSA)(Sigma-Aldrich), followed by 20 min washout in Ringer solution without BSA, supplemented with 30 µM N-benzyl-p-toluene sulfonamide (BTS, Tocris) to prevent fiber contraction.

Total releasable Ca^2+^ store content was indirectly determined, by measuring cytosolic Ca^2+^ release induced by treatment with depletion cocktail (ionomycin-CPA-EGTA; ICE) composed by 10 µM ionomycin (ThermoFisher Scientific) and 30 µM cyclopiazonic acid (CPA) in calcium free Ringer solution, containing 0.2 mM EGTA (Hanna et al. 2021; Michelucci et al. 2022). Ca^2+^ measurements were performed using a Nikon Diaphot 300 inverted microscope (Nikon) equipped with a CCD camera (Photometrics, Roper Scientific) and a MetaFluor imaging system (Universal Imaging). Fluorescence images were collected with a fluor 20X objective (Nikon). The use of GFP was essential for selecting muscle fibers expressing recombinant CASQ1 and conducting ex vivo studies. Damaged fibers and either fibers showing GFP overexpression or low-expression levels of GFP were excluded from analysis. In performing these experiments, the GFP fluorescence of GFP-CASQ1 fusion proteins was evaluated by MetaMorph acquisition software. The fluorescence intensity of single fibers was acquired with an exposure time of one second. Fibers with a fluorescence intensity in the range between 4000 and 6000 arbitrary units, measured according to the MetaMorph software, were selected for further Ca^2+^ imaging analysis. To assess the Ca^2+^ store content, the Area Under the Curve (AUC) of fluorescence ratio acquired at 340/380 nm within 6 min following the addition of the ICE depletion cocktail was calculated.

### Mn^2+^ quench to measure SOCE

For Mn^2+^ quench studies, Fura-2AM loaded FDB muscle fibers were analyzed in Ca^2+^-free Ringer solution added with BTS and excited at 360 nm (isobestic point of Fura-2), while emission was detected at 510 nm. After establishing the baseline rate of Fura-2 decay, fibers were exposed to a Ca^2+^-free Ringer solution supplemented with 0.5 mM MnCl_2_. The maximum rate of change in Fura-2 fluorescence in the presence of Mn^2+^ (referred to as Rate_max_) was determined from the peak time derivative of the Fura-2 emission trace during Mn^2+^ application. The maximum rate of SOCE was calculated as Rate_SOCE_ = Rate_max_ − Rate_baseline_ and expressed as the change in fluorescence per unit time (dF/dt) in seconds, following previously established methods (Hanna et al. 2021; Michelucci et al. 2022).

### Localization of CASQ1 and immunofluorescence on FDB muscle fibers

Isolated FDB fibers were plated on laminin-coated glass bottom dishes and in 4% paraformaldehyde (PFA), 2% sucrose, 0.5% Triton X-100 diluted in Phosphate Buffer Saline (PBS) (137 mM NaCl, 2.7 mM KCl, 4.3 mM Na_2_HPO_4_, 1.4 mM KH_2_PO_4_) for 1 h at room temperature. The bundles of fibers were further permeabilized with Hepes-Triton-X 100 buffer (20 mM Hepes pH 7.4, 300 mM sucrose, 50 mM NaCl, 3 mM MgCl_2_, 0.5% Triton X-100) for 3 min. Fibers were blocked in 5% goat serum, 2% BSA, 0.1% Triton X-100 in PBS for 1 h. To identify triads, rabbit polyclonal antibodies against RyR1 (Rossi et al. 2014a) and AlexaFluor555 anti-rabbit secondary antibodies (Thermo-Scientific) were used for immunofluorescence detection. Anti-RyR1 antibodies were generated in our laboratories and are directed against the divergency region 1 of mouse RyR1 (residues 4418–4562 in sequence Uniprot code A0A1L1SQG7). Primary antibodies were prepared in 2% BSA and incubated overnight at 4 °C. Slides were mounted with Mowiol (Mowiol 4–88, Sigma-Aldrich, 20% diluted in PBS) and analyzed with an LSM-510 META confocal microscope (ZEISS). Analysis of co-localization was performed using the Plot profile and Just Another Colocalization (JACoP) plugins of the ImageJ software (https://imagej.net).

### Statistical analysis

**Fig. 1:**
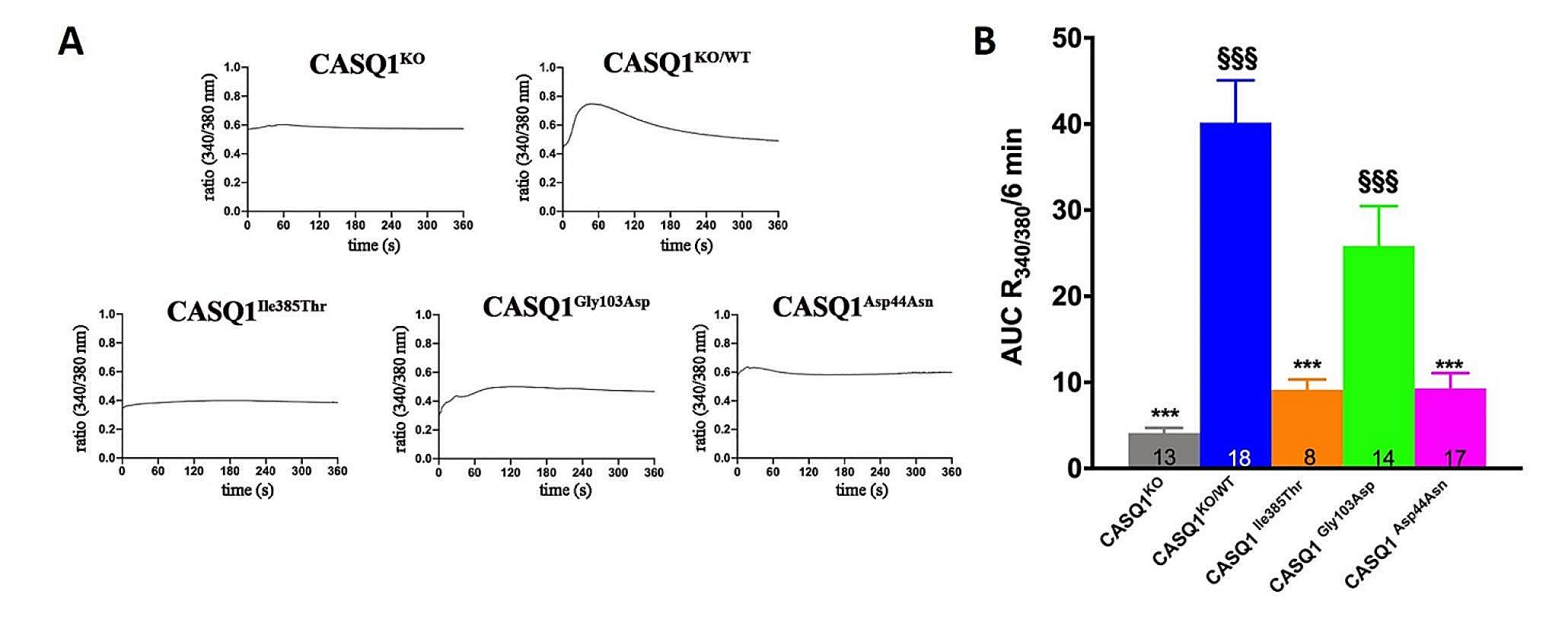
Evaluation of Ca^2+^ store content in isolated FDB muscle fibers. Ca^2+^ store content was analyzed in FDB muscles from *Casq1* knockout mice and in fibers expressing CASQ1WT-GFP and CASQ1-GFP mutants following treatment with ICE store depletion medium. (a) Representative traces of Fura-2 fluorescence ratio of 340/380 nm, starting from ICE addition, and quantitative analyses of total releasable Ca^2+^ store content (b). Numbers on bars indicate the number of fibers tested for each sample. Experiments were conducted using 3 to 5 male mice. Bars indicate mean values of AUC ± SEM. Statistically significant differences compared to fibers expressing CASQ1^WT^ are indicated by * (*p* values ≤ 0.05), ** (*p* values ≤ 0.01), *** (*p* values ≤ 0.001). Statistically significant differences compared to FDB fibers from *Casq1* knockout mice are indicated by §§§ (*p* values ≤ 0.001)

**Fig. 2:**
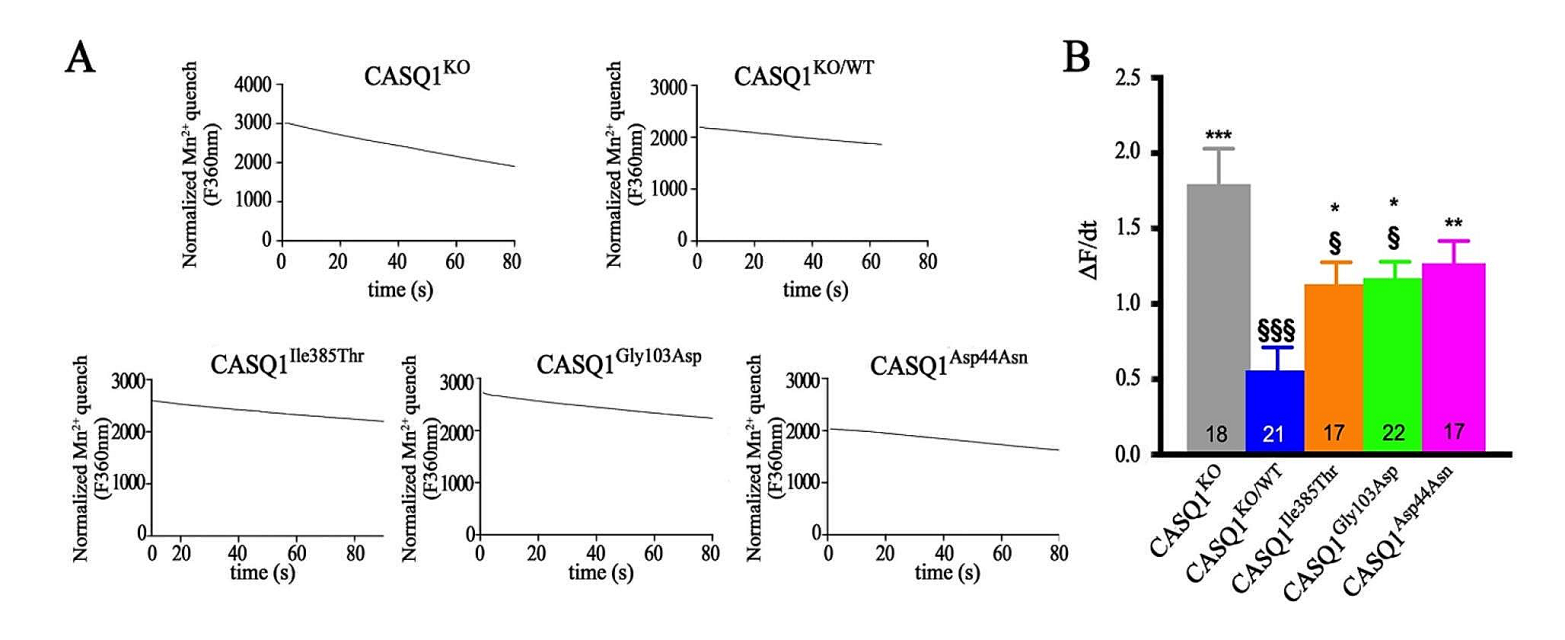
Evaluation of SOCE in isolated FDB muscle fibers. SOCE was analyzed in FDB muscle fibers from *Casq1* knockout mice and in fibers expressing CASQ1WT-GFP and CASQ1 mutants. (a) Representative traces of maximal rate of Fura-2 fluorescence quench by Mn^2+^, normalized for the initial baseline rate of Fura-2 decay, starting with the application of 0.5 mM Mn^2+^ in FDB fibers. (b) Bars indicate mean dF/dt values ± SEM. Numbers on bars indicate the number of fibers tested for each sample. Experiments were conducted using 3 to 5 male mice. Statistically significant differences compared to fibers expressing CASQ1^WT^ are indicated by * (*p* values ≤ 0.05), ** (*p* values ≤ 0.01), *** (*p* values ≤ 0.001). Statistically significant differences compared to FDB fibers from *Casq1* knockout mice are indicated by § (*p* values ≤ 0.05), §§§ (*p* values ≤ 0.001)

Statistical analyses and graphical presentation were made by Prism 7 (GraphPad Software). Statistical analysis was performed by the non-parametric Kruskal-Wallis test with Dunn’s multiple comparison correction (Fig. 1) and by ordinary one-way ANOVA with Dunnett’s multiple comparisons test (Fig. 2). Normality test was performed by the Kolmogorov-Smirnov, D’Agostino and Pearson normality tests. Differences were considered as statistically significant at *, §, *p* < 0.05. Data were presented as mean ± SEM.

## Results

### Expression of CASQ1 proteins carrying mutations associated with TAM partially restores SR Ca^2+^ store content in Flexor Digitorum Brevis (FDB) muscles from *Casq1 *knockout mice

**Fig. 3:**
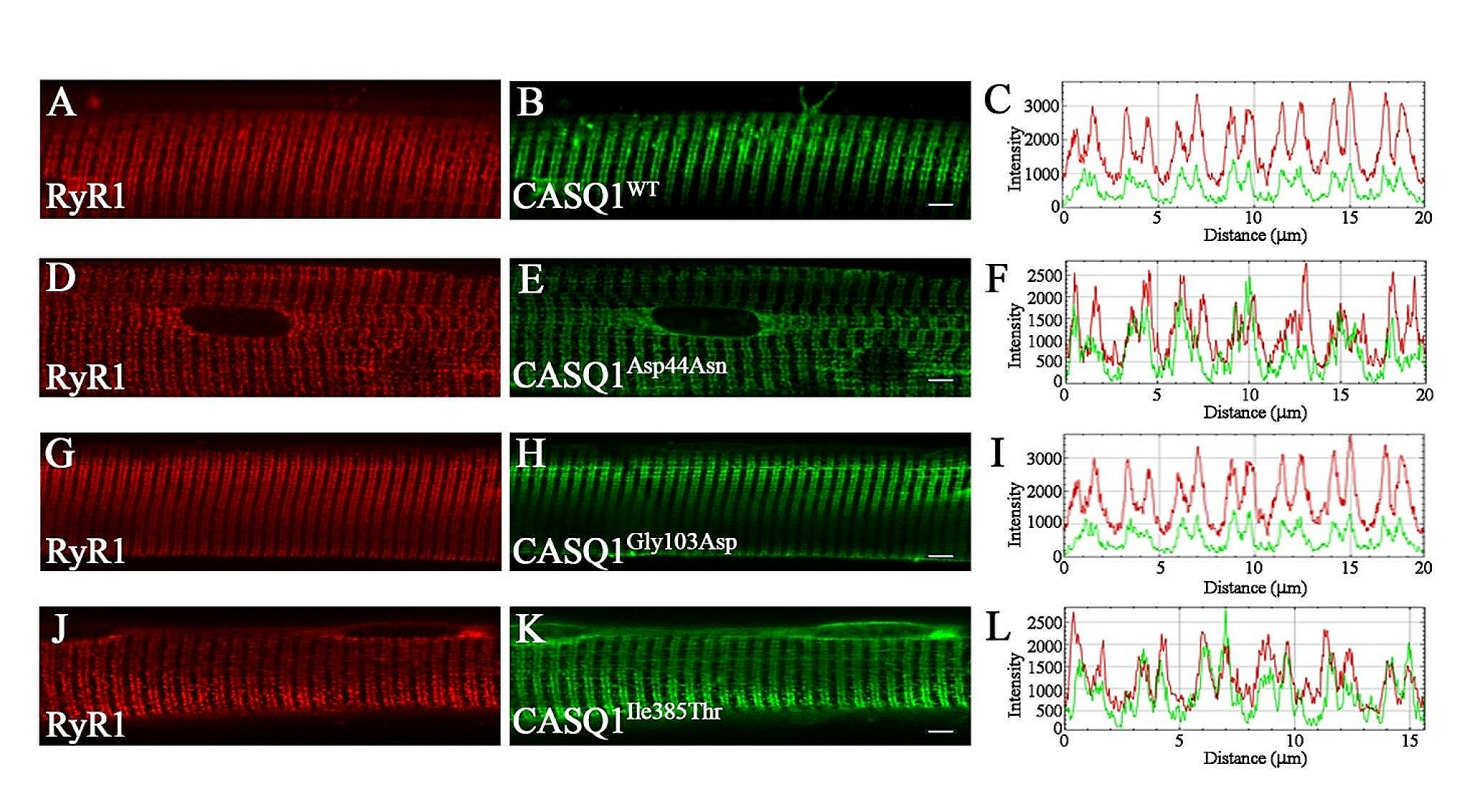
Expression of CASQ1WT and CASQ1 mutants in FDB from *Casq1* knockout mice. Representative FDB muscle fibers isolated from *Casq1* knockout mice transfected with CASQ1^WT^ (B), CASQ1^Asp44Asn^ (E), CASQ1^Gly103Asp^ (H), CASQ1^Ile385Thr^ (K). FDB muscle fibers were stained with rabbit primary antibodies against RyR1 and Alexa Fluor 555-conjugated anti rabbit secondary antibodies to label triads (A, D, G and J). To evaluate colocalization, the fluorescence intensity of RyR1 (red) and CASQ1 (green) was plotted as a function of distance for six to seven successive triads (C, F, I and L). Pearson coefficients of co-localization range from 0.712 to 0.741. Scale bar = 3 μm

*Casq1* knockout mice show a significant decrease in the SR Ca^2+^ content and impairment in sustained muscle activity (Paolini et al. 2007; Tomasi et al. 2012). Interestingly, re-expression of CASQ1 in FDB muscles from *Casq1* knockout mice resulted in rescue of peak amplitude of Ca^2+^ transients following electrical stimulation to values comparable to those of wild type fibers (Tomasi et al. 2012). These results indicate that the *Casq1* knockout mouse represents a valuable model to evaluate the effect of the CASQ1^Asp44Asn^, CASQ1^Gly103Asp^ and CASQ1^Ile385Thr^ on skeletal muscle fiber Ca^2+^ homeostasis. Accordingly, we expressed wild type and mutant CASQ1-GFP tagged proteins in FDB muscles from *Casq1* knockout mice. Confocal laser scan microscopy analysis of isolated fibers showed that both wild type and mutant CASQ1-GFP tagged proteins selectively localized at triads, with a pattern that was indistinguishable from that obtained by staining fibers with antibodies against RyR1, the calcium release channel of the triad (Fig. 3A-K).

To evaluate the total releasable SR Ca^2+^ content of muscles expressing TAM-associated CASQ1 mutations, isolated FDB fibers were treated with ICE depletion solution and cytosolic Ca^2+^ increase was measured in the following 6 min and expressed as Area Under the Curve (AUC) (Fig. 1). Ca^2+^ imaging was performed on fibers meticulously selected after eliminating those with either overexpression or too low-expression levels of GFP. Only fibers expressing intermediate levels of GFP fluorescence were selected for further analysis.

Expression of CASQ1^WT^ in *Casq1* knockout mice resulted in an increase of the SR releasable Ca^2+^ content of about 9.75 times compared to *Casq1* knockout mice, indicating that expression of recombinant CASQ1 recovers the intracellular Ca^2+^ store content of FDB fibers (40.16 ± 4.91 and 3.62 ± 0.37 arbitrary units in fibers expressing CASQ1^WT^ and *Casq1* knockout fibers, respectively). On the contrary, expression of neither one of the CASQ1 mutants results in an increase in the intracellular Ca^2+^ content comparable to that observed with CASQ1^WT^, confirming that the three mutations in CASQ1 alter the Ca^2+^ binding properties of the protein (9.3 ± 1.76, 25.83 ± 4.62, 9.15 ± 1.18 arbitrary units in fibers expressing CASQ1^Asp44Asn^, CASQ1^Gly103Asp^ and CASQ1^Ile385Thr^, respectively). Only the expression of the CASQ1^Gly103Asp^ mutant can partially recover the intracellular Ca^2+^ store content of *Casq1* knockout fibers, although at levels non statistically significant compared to those measured following expression of CASQ1^WT^ (Fig. 1).

### Expression of CASQ1^WT^, but not of CASQ1 proteins carrying mutations associated with TAM, inhibits SOCE in FDB muscles from *Casq1 *knockout mice

The depletion of ER/SR Ca^2+^ content triggers the activation of SOCE, allowing the influx of Ca^2+^ from the extracellular space to restore Ca^2+^ stores (Roos et al. 2005; Feske et al. 2006). Given that *Casq1* knockout mice exhibit a reduced luminal Ca^2+^ content due to the absence of CASQ1, they display a constitutively active SOCE to prevent excessive and dangerous depletion during muscle contraction (Michelucci et al. 2022).

We thus employed *Casq1* knockout mice as a model to assess the impact of CASQ1 mutations associated with TAM on SOCE inhibition (Fig. 2). As expected, the expression of CASQ1^WT^ in FDB muscle fibers led to a significant decrease of SOCE, with an inhibition of about 3.2 times compared to fibers from *Casq1* knockout mice (0.56 ± 0.15 and 1.79 ± 0.24 arbitrary units in fibers expressing CASQ1^WT^ and *Casq1* knockout fibers, respectively). Conversely, neither one of the CASQ1 mutants induced a significant SOCE inhibition, suggesting that, beyond altering Ca^2+^ binding, CASQ1 mutations also influence SOCE regulation (1.27 ± 0.15, 1.17 ± 0.11 and 1.13 ± 0.14 arbitrary units in fibers expressing CASQ1^Asp44Asn^, CASQ1^Gly103Asp^ and CASQ1^Ile385Thr^, respectively).

## Discussion

TAM was originally described as a myopathy associated with gain of function mutations in *STIM1* and *ORAI1*, the two key proteins participating in the SOCE mechanism. Muscle fibers from patients or mouse models of TAM show an increase in Ca^2+^ entry due to constitutive ORAI1 channel activation and excessive Ca^2+^ entry is proposed as a major cause for tubular aggregates formation (McCarl et al. 2009; Böhm et al. 2013; Misceo et al. 2014; Nesin et al. 2014; Lacruz and Feske 2015; Böhm et al. 2017; Böhm and Laporte 2018; Choi et al. 2019; Silva-Rojas et al. 2020). In the last decade, mutations in *CASQ1* have been identified in patients diagnosed with Vacuolar Myopathy and TAM (Rossi et al. 2014b; Lewis et al. 2015; Barone et al. 2017; Böhm et al. 2018b; Hanna et al. 2021). Characterization of CASQ1 mutants demonstrated that they all display a significant alteration in Ca^2+^ dependent polymerization that, at the functional level, results in a reduction in Ca^2+^ storage capacity (Rossi et al. 2014b; Lewis et al. 2015; Barone et al. 2017; Böhm et al. 2018b; Hanna et al. 2021). CASQ1 was also reported to bind both STIM1 and STIM2 and to inhibit SOCE (Wang et al. 2015; Zhang et al. 2016; Jeong et al. 2021). Accordingly, analysis of the TAM associated mutants CASQ1^Asp44Asn^ and CASQ1^Ile385Thr^ expressed in HeLa cells, showed that these mutants not only exhibit alterations in Ca^2+^ storage, but they also elicit an increase in Ca^2+^ entry following ER store depletion, suggesting that those alterations observed in muscles from patients carrying these mutations may indeed correlate with a perturbation in Ca^2+^ balance (Barone et al. 2017; Böhm et al. 2018b). To support this idea, we expressed GFP-tagged mutants CASQ1^Asp44Asn^, CASQ1^Gly103Asp^ and CASQ1^Ile385Thr^ in muscles from *Casq1* knockout mice to replicate the cellular milieu where CASQ1 is naturally expressed. We focused on FDB muscle due to its typically high electroporation efficiency and because it is rich in fast-twitch oxidative-glycolytic (type IIA) fibers, where the influence of CASQ2 on Ca^2+^ homeostasis is minimal. Although mutants are not expressed at the homozygous state in TAM patients, *Casq1* knockout mice still represent a valuable model to functionally characterize CASQ1 mutants. Indeed, it was previously shown that recombinant CASQ1 expressed in muscle fibers of *Casq1* knockout mice can correctly target to the junctional SR and rescue Ca^2+^ homeostasis at levels comparable to those observed in wild type mice (Tomasi et al. 2012). Confocal laser scan microscopy analysis on FDB muscle fibers from *Casq1* knockout mice transiently expressing CASQ1^Asp44Asn^, CASQ1^Gly103Asp^ and CASQ1^Ile385Thr^ showed that, similarly to what previously observed with CASQ1^Asp244Gly^, all mutans localized at the junctional SR; this indicates that, despite their altered Ca^2+^ dependent polymerization, targeting at triads is not impaired (Barone et al. 2017).

Changes in CASQ1 protein content are commonly associated with alteration in intraluminal Ca^2+^ content (Paolini et al. 2007; Boncompagni et al. 2012; Marabelli et al. 2023; Watanabe et al. 2024). As previously mentioned, re-expression of CASQ1 in muscles from *Casq1* knockout mice can restore intraluminal Ca^2+^ to levels comparable to those observed in wild type mice (Tomasi et al. 2012). Conversely, when measuring intracellular Ca^2+^ levels in FDB muscle fibers expressing CASQ1 mutants, it was observed that none of these mutants could restore the releasable SR Ca^2+^ store content to levels comparable to those measured in fibers expressing recombinant wild-type CASQ1, confirming that they all display a significant reduction in Ca^2+^ binding capacity. Only CASQ1^Gly103Asp^ maintains a relative capacity to support luminal Ca^2+^ store content, compared to CASQ1^Asp44Asn^ and CASQ1^Ile385Thr^ mutants. This agrees with evidence that at elevated Ca^2+^ concentrations, CASQ1 mutants show polymerization kinetics different from those observed with wild type CASQ1 (Barone et al. 2017; Böhm et al. 2018b). In addition, results from experiments performed in HeLa cells and in C2C12 myoblasts showed that another CASQ1 mutant, CASQ1^Asn56Tyr^, and CASQ1^Gly103Asp^ have a lower propensity to depolymerize upon treatment with the SERCA inhibitor thapsigargin (Barone et al. 2017; Böhm et al. 2018b). This observation suggests a mechanism that may further contribute to explain the decreased levels of intracellular Ca^2+^ content measured in FDB muscle fibers expressing TAM-associated CASQ1 mutants.

*Casq1* knockout mice also represent a valuable model to study Ca^2+^ entry, since muscle fibers from these mice show a constitutively active SOCE to sustain muscle fatigue in the absence of a proper intracellular Ca^2+^ store (Michelucci et al. 2022). Studies aimed at investigating the ability of CASQ1 mutants to inhibit SOCE revealed that all three mutants lost the ability to inhibit Ca^2+^ influx in *Casq1* knockout muscle fibers at levels comparable to wild type CASQ1. Only the CASQ1^Asp44Asn^ mutant showed levels of Ca^2+^ entry not statistically different from that of *Casq1* knockout muscle fibers, indicating that this mutation appears to display more severe effects on SOCE. Interestingly, mice knock-in for the CASQ1^Asp244Gly^, the CASQ1 mutation identified in patients with vacuolar myopathy, did not display any difference in SOCE compared to wild type mice (Rossi et al. 2014b). This indicates that the ability to affect SOCE is a distinctive trait of CASQ1 mutations associated with TAM.

SOCE inhibition is mediated by direct binding of CASQ1 to both STIM1 and STIM2. Biochemical and functional studies showed that region encompassing amino acids 7 to 92 in mouse CASQ1 binds the STIM2 isoform; accordingly, expression of this region in mouse primary myoblasts results in SOCE inhibition (Jeong et al. 2021). On the contrary, amino acid residues 362–387 in human CASQ1 bind to STIM1 (Wang et al. 2015; Zhang et al. 2016). However, the precise mechanisms underlying SOCE inhibition mediated by CASQ1 have not been completely unraveled and require further experiments. Regarding CASQ1 mutants, our previous data indicate that the expression of these mutants in HeLa cells led to a reduction in SOCE inhibition. This reduction could not be attributed to altered STIM1 recruitment to the plasma membrane or to altered STIM1 clustering (Barone et al. 2017), suggesting that a different mechanism, potentially also present in skeletal muscle fibers, is involved. However, it is essential to note that the mouse model used in this study, at difference with HeLa cells, exhibits a constitutively active SOCE in part due to diminished SR Ca^2+^storage resulting from CASQ1 deficiency, and in part due to the constitutive assembly of Ca^2+^ entry units. Re-expression of wild-type CASQ1 in muscle fibers from *Casq1* knockout mice induced a partial, yet significant regression of the ultrastructural sarco-tubular alterations observed in the *Casq1* knockout mouse model and restored SR Ca^2+^ levels (Tomasi et al. 2012). Unfortunately, no information is provided regarding the regression of Ca^2+^ entry units upon re-expression of mutant CASQ1. Therefore, it is essential to acknowledge that in our experimental model, multiple factors may contribute to the reduction of SOCE inhibition observed following mutant CASQ1 expression. These factors may include direct effects on STIM1 or STIM2, as well as indirect effects on the number of Ca^2+^ entry units, potentially influencing SOCE inhibition. Future investigations will help to extend our knowledge concerning these aspects.

In conclusion, results obtained following expression of CASQ1 mutants in FDB muscle fibers from *Casq1* knockout mice suggest that different mechanism can cooperate to trigger the development of TAM in patients with CASQ1 mutations: on one side the lower binding capacity of the three CASQ1 mutants that directly affects SR Ca^2+^ storage, and, on the other side, the alteration in regulation of Ca^2+^ entry that contribute to deregulate Ca^2+^ homeostasis (Barone et al. 2017).

These results collectively support the notion that STIM1, ORAI1, and CASQ1 are components of the same cellular pathway regulating SOCE in skeletal muscle and that alteration in Ca^2+^ entry represents a hallmark of TAM. This hypothesis is reinforced by evidence obtained from the characterization of several murine knock-in mouse models carrying mutations found in TAM patients. Indeed, although not all characteristic phenotypes of the human disease can be reproduced in these murine models of TAM, altered and increased SOCE is a constant finding (Grosse et al. 2007; Cordero-Sanchez et al. 2019; Silva-Rojas et al. 2019, 2021; O’Connor et al. 2023). Accordingly, approaches aimed at inhibiting or downregulating SOCE, independently from the gene/mutation identified in patients, may reveal as a successful therapeutic strategy to partially antagonize the TAM phenotype (Feske et al. 2006; Luo et al. 2019; Riva et al. 2022; Silva-Rojas et al. 2022, 2024; Cordero-Sanchez et al. 2022).

Moreover, from a diagnostic perspective, the finding that mutations found in either *STIM1*, *ORAI1* or *CASQ1* all alter SOCE suggests that extending genetic analysis to other genes involved in the regulation of SOCE, may potentially improve patients’ diagnosis and stratification. Further support to this hypothesis comes from studies on some non-TAM myopathies, that might also occasionally present tubular aggregates in muscle biopsies. These myopathies, although caused by mutations in genes associated with the regulation of the N-linked glycosylation pathway, also seem to involve pathways comprising proteins participating in the regulation of Ca^2+^ homeostasis, including STIM1 (Funk et al. 2013; Bauché et al. 2017; Gang et al. 2022; Brande et al. 2023). Future work will help to further enhance our comprehension of the mechanisms underlying TAM development and the potential role of STIM1 and ORAI also in other myopathies.

## Data Availability

No datasets were generated or analysed during the current study.
